# Prophylactic antibiotic bundle compliance and surgical site infections: an artificial neural network analysis

**DOI:** 10.1186/s13037-019-0222-4

**Published:** 2019-12-07

**Authors:** Steven Walczak, Marbelly Davila, Vic Velanovich

**Affiliations:** 1 School of Information and Florida Center for Cybersecurity, University of South Florida, Tampa, FL USA; 2 College of Business, Information and Technology Management, University of Tampa, 5 Tampa General Circle, Suite 740, Tampa, FL 33606 USA; 3 Tampa General Hospital, Tampa, FL USA; 4 Division of General Surgery, University of South Florida, Tampa, FL USA

**Keywords:** Surgical site infection, Prophylactic antibiotic bundle compliance, Artificial neural networks

## Abstract

**Background:**

Best practice “bundles” have been developed to lower the occurrence rate of surgical site infections (SSI’s). We developed artificial neural network (ANN) models to predict SSI occurrence based on prophylactic antibiotic compliance.

**Methods:**

Using the American College of Surgeons National Quality Improvement Program (ACS-NSQIP) Tampa General Hospital patient dataset for a six-month period, 780 surgical procedures were reviewed for compliance with SSI guidelines for antibiotic type and timing. SSI rates were determined for patients in the compliant and non-compliant groups. ANN training and validation models were developed to include the variables of age, sex, steroid use, bleeding disorders, transfusion, white blood cell count, hematocrit level, platelet count, wound class, ASA class, and surgical antimicrobial prophylaxis (SAP) bundle compliance.

**Results:**

Overall compliance to recommended antibiotic type and timing was 92.0%. Antibiotic bundle compliance had a lower incidence of SSI’s (3.3%) compared to the non-compliant group (8.1%, *p* = 0.07). ANN models predicted SSI with a 69–90% sensitivity and 50–60% specificity. The model was more sensitive when bundle compliance was not used in the model, but more specific when it was. Preoperative white blood cell (WBC) count had the most influence on the model.

**Conclusions:**

SAP bundle compliance was associated with a lower incidence of SSI’s. In an ANN model, inclusion of the SAP bundle compliance reduced sensitivity, but increased specificity of the prediction model. Preoperative WBC count had the most influence on the model.

## Background

Surgical site infections (SSI’s) are a common problem after many types of operations. Approximately 2% of all patients who undergo an operation in the United States will develop an SSI [1, 2], with some types of operations, such as colonic surgery, being much higher [3]. In addition to the suffering this causes patients, it is a substantial financial burden [4]. This has led to efforts to identify risk factors for SSI’s and to reduce the incidence of SSI.

There are numerous risk factors for the development of SSI’s. The July, 2017 American College of Surgeons National Quality Improvement Program (ACS-NSQIP) SSI model identified 28 statistically significant risk factors [5]. These include male sex, age, race, type of operation, operation complexity, American Society of Anesthesiologists’ (ASA) classification, obesity/underweight, smoking status, inpatient surgery, wound classification, preoperative sepsis, surgical specialty, steroid use, diabetes mellitus, sodium level, chronic obstructive pulmonary disease, alkaline phosphatase level, functional status, bleeding disorders, weight loss, ventilator dependence, albumin level, dyspnea, creatinine level, hypertension, and thrombocytosis. Although these are identified as individual risk factors, how they interact with each other to potentiate or mitigate the risk for an individual patient is not well known.

General approaches to reducing SSI’s have been to improve the individual patient’s risk profile or to apply specific practices to mitigate risk. For example, Alexander, et al. [6] recommended an aseptic operating room environment, preoperative antiseptic bathing, hair removal by clipping, skin decontamination, drapes, bacterial-resistant suture, prophylactic antibiotics, maintenance of normal body temperature, maintenance of optimal oxygen saturation, glucose control, minimizing blood transfusions, judicious fluid management and, when necessary, delayed primary closure [6]. The value of each of these recommendations, let alone their use in combination, is variable.

There have been numerous attempts to create protocols to reduce the incidence of SSI’s. One of the earliest was the Surgical Care Improvement Program (SCIP), which put forth guidelines aimed at SSI reduction [7]. However, the practical results of SCIP implementation have been mixed at best [8]. This has led others to develop SSI reduction strategies based on evidence-based best practices. Some of these SSI reduction “bundles” have had more success [9], especially in more high-risk operations such as colorectal surgery [10, 11].

The American College of Surgeons National Surgical Quality Improvement Program (ACS-NSQIP) allows hospitals to obtain risk-adjusted data of their outcomes, including SSI. The purpose is to provide hospitals with reliable data that they can use to implement quality improvement initiatives. ACS-NSQIP provides best practice guidelines to help with these quality improvement initiatives, including one for SSI (Table 1) [12]. Nevertheless, because the occurrence of SSI is complex with only a few factors in the surgeon’s control, we hypothesized that there was more to this phenomenon.

**Table 1:** ACS-NSQIP Best Practice SSI Prevention Bundle

Preoperative	Intraoperative	Postoperative
Patient-Related	Patient-Related	Patient-Related
Encourage patient to discontinue tobacco use for at least 30 days prior to operation	Monitor and maintain glucose levels (< 200 mg/dl) in cardiothoracic surgery patients, including non-diabetic patients	Monitor and maintain glucose levels (< 200 mg/dl) in cardiothoracic surgery patients, including non-diabetic patients
Identify and treat all non-surgical site infections prior to surgery. Postpone elective operations if necessary	Discontinue prophylactic antibiotics within 24 h after noncardiac surgery and 48 h after cardiac surgery	Discontinue prophylactic antibiotics within 24 h after noncardiac surgery and 48 h after cardiac surgery
Administer prophylactic antibiotics within one hour prior to surgery (vancomycin and fluoroquinolones should be administered two hours prior to surgery). Select the appropriate antimicrobial prophylaxis based on evidence-based guidelines	Cover primarily closed incisions with a sterile dressing for 24 to 48 h postoperatively. Wash hands before and after any contact with surgical site.	Cover primarily closed incisions with a sterile dressing for 24 to 48 h
Adjust the dose of the prophylactic antibiotics for morbid obesity		
Provider-Related	Provider-Related	
Keep nails short. Do not wear artificial nails or hand or arm jewelry	Wear a cap or hood to fully cover head/facial hair and a surgical mask to cover nose/mouth when entering the operating room (if operation is about to begin, is underway, or surgical instruments are exposed) and until the conclusion of the operation.	
Clean underneath fingernails prior to first daily surgical scrub. Complete a two to five minute preoperative scrub using appropriate antiseptic or use alcohol-based surgical antiseptic	Use surgical gown and drapes that are liquid resistant	
	Wear sterile gloves if a scrubbed surgical team member	
	Change surgical scrubs if grossly soiled or contaminate	
	System-Related	
	Comply with standards regarding operating room asepsis	
	When visible contamination of surfaces/equipment with body fluids occurs, use an Environmental Protection Agency-approved cleaning solution to clean affected area before next operation	

Artificial neural networks (ANNs) are a nonparametric machine learning method based on modeling the neuronal activity of the human brain. Neuronal activity is simulated using processing elements referred to as neurodes that are arranged in layers and connected to neurodes in subsequent layers through a connection which carries a weight value. The weighted values of the connections indicate the strength of the neuronal signal from one neurode to the next. ANNs can develop predictive models based on the program’s ability to “learn” through adjustment of the weight values on the interlayer connections. ANN learning enables the ANN to accurately model nonlinear relationships between factors related to a clinical problem and the clinical problem’s results. We chose ANN as the machine learning method explicitly because of its ability to identified “hidden” relationships in data sets that may not be obvious through standard, linear models.

We suspected that in addition to the surgical antimicrobial prophylaxis (SAP) bundle compliance, other factors may also drive SSI occurrences. Our premise is that the occurrence of a SSI is a complex process, due to the interaction of numerous patient risk factors and patient-care process factors that occur with each operation. Our aim is to apply ANNs to SSI occurrence across surgical specialties to determine the efficacy of ANN models to predict SSI occurrence and to assess the influence of ACS-NSQIP compliance in predicting SSI occurrence. Our hypothesis is that SAP bundle compliance will reduce the incidence of SSI occurrences and be predictive of an SSI-free surgical outcome.

## Methods

This study was approved by the institutional review boards of the University of South Florida and Tampa General Hospital.

All patients who underwent operations at Tampa General Hospital, a large, urban, tertiary, teaching hospital located in Tampa, Florida, USA, and were subsequently submitted to the ACS-NSQIP data base and reported back to Tampa General Hospital from 1 July 2015 to 31 December 2015 were included in this study. The primary outcome measure was SSI occurrence, and the secondary outcome measure was SAP compliance. Patients who underwent operations and whose data were not submitted to the ACS-NSQIP data base or were outside this time frame were excluded. Use of the ACS-NSQIP definitions for patient risk factors and SSI occurrence was done to have uniform definitions of risk variables and SSI’s. A total of 780 distinct records were obtained for analysis. Once this list was generated, variables including age, sex, operation, surgeon, surgical specialty and wound classification were collected for SSI occurrence analysis. These variables were chosen because they could be consistently found in the records of all patients and would provide a profile of the breath of operative experience at Tampa General Hospital. Specifically, for surgeon and surgical specialty, we wished to identify if individual surgeons or surgical specialty, as they generally adhered to similar practice patterns, influence SSI occurrences. The records of these patients were reviewed for adherence to the ACS-NSQIP best practice SSI prevention bundle [12]. Table 1 presents the entire ACS-NSQIP recommend SSI reduction bundle. We specifically determined whether two aspects of the bundle were adhered to: the type of antibiotic and timing of administration. Other aspects of the bundle could not be assessed due to lack of pertinent information in the record. These data would provide the basis of the incidence of SSI occurrences in patients compliant and non-compliant to the SAP bundle.

### Statistical analysis

We compared the group of patients where both the type of antibiotic and the timing of the antibiotic (SAP bundle) were compliant with the ACS-NSQIP SSI reduction recommendations to the group where they were not compliant. This was the basic determination if compliance was associated with lower SSI rates. Variability of antibiotic timing was also determined. Categorical data were described as totals, frequencies, and percentages; continuous variables were described as means with standard deviation and medians with interquartile ranges. Univariate comparisons were assessed for covariates and outcome variable between SAP compliant and SAP noncompliant applying the chi-square test or Fisher’s exact test for categorical variables. A *p*-value of 0.05 or less was considered statistically significant. Data were analyzed with SAS 9.4 software, SAS Institute Inc., Cary, NC, USA. For this portion of the analysis, all 780 patient records were used.

### Artificial neural network analysis

ANNs have been shown to be universal approximators for arbitrarily complex, including nonlinear, problems [13], and thus ANN models were developed to predict SSI occurrence. The 11 independent variables analyzed were: age (in years), sex (male/female), steroid use (present/not present), bleeding disorders (present/not present), transfusion (present/not present), white blood cell count (WBC/mm^3^), hematocrit level (%), platelet count (platelets/mm^3^), wound classification (class 1, 2, 3, or 4), ASA class (class 1, 2, 3, 4, or 5), and NSQIP surgical antimicrobial prophylaxis bundle compliance (present/not present). The dependent variable was the occurrence of an SSI, which is represented with a value 1 for an SSI and a value − 1 for no SSI. As ANN can only analyze complete data sets, surgical population sample data with missing values were eliminated from the ANN training and evaluation data. A total of 646 clean records were used for training and validating the ANNs.

The training algorithm used for the ANN models was backpropagation, which is the most commonly used training algorithm in medical ANN models [14, 15]. Various architectures were designed following best practices [16, 17] using both single hidden layer and two hidden layer designs, with various quantities of neurodes per hidden layer. For each architecture for each of the ANN models, 3-fold cross validation was used to separate training and validation data samples. The two hidden layer architectures performed the best and are the ones reported below.

**Fig. 1:**
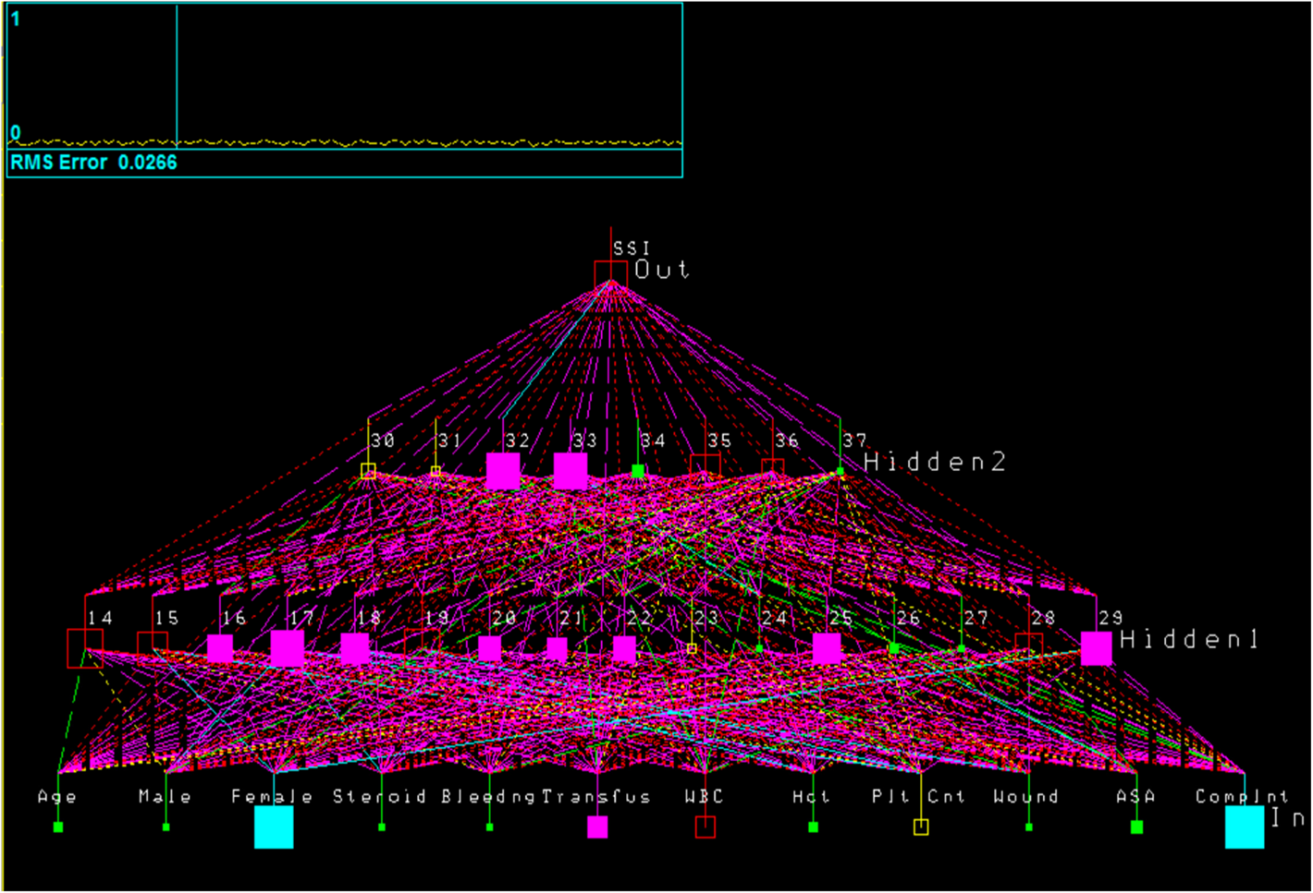
SSI Prediction Artificial Neural Network Architecture (w/ bundle compliance)

Two distinct ANN models were developed using the NeuralWare® NeuralWorks Professional II Plus© neural network shell tool. The first ANN model included the NSQIP SAP bundle compliance variable and the second ANN model used the other 10 variables without the NSQIP SAP bundle compliance variable. The optimal architecture for the full 11 variables ANN model is presented in Fig. 1 (the other ANN architectures are similar). The purpose of the distinct ANN models is to evaluate the importance and influence of specific variables using the leave-one-variable-out method, which is a commonly utilized technique for determining variable influence in ANNs [18, 19]. One additional model was developed that also left out the sex variable, in addition to the bundle compliance variable, to evaluate its influence on SSI predictions.

### Determination of relative importance

After all the different ANN architectures and models were evaluated, one further ANN model that duplicated the complete 11 variable set was developed using the JustNN© neural network shell tool to further evaluate variable contributions to SSI prediction. JustNN© tries to determine variable influence using the sum of the weighted connections between processing elements in the ANN [20].

## Results

A total of 780 surgical procedures from 9 surgical specialties including 112 surgeons and 220 distinct CPT coded operations were collected. Table 2 presents the patient demographic information and SSI outcome stratified by SAP bundle compliance status. Over 60% of the patients were female, and the range of ages was from 19 to 94 years old. The distribution of wound classification was clean 55.0%, clean-contaminated 37.8%, contaminated 2.8%, and dirty 4.4%. The distribution of surgical specialties was general surgery 40.4%, gynecology 16.3%, neurosurgery 12.1%, orthopedic surgery 11.9%, urology 8.6%, vascular surgery 5.6%, plastic surgery 1.9%, thoracic surgery 1.9% and otolaryngology 1.2%. Individual surgeon data was not analyzed as there were too few operations for each surgeon to make for a meaningful analysis. The overall compliance of the prophylactic antibiotic SAP bundle was 92.0% (718 of 780 patients), with appropriate antibiotic compliance of 94.6% (738 of 780 patients) and appropriate timing of 92.1% (719 of 780 patients). Of note, patients in the non-compliant group were more frequently smokers and underwent emergent operations, while patients in the compliant group were more frequently undergoing elective operations (Table 2). There were no other statistically significant differences between the groups. Table 3 shows the mean time of antibiotic administration to surgical incision based on specialty with the 25th, 50th percentile and 75th percentile times of the time distribution. The median time to administration was within 7 min among the specialties. Three specialties had fourth quartile times of greater than 30 min, while one had fourth quartile time of less than 20 min. The distribution of SSI’s types was superficial 31% (9 of 29 patients), deep 38% (11 of 29 patients) and organ space 31% (9 of 29 patients). Overall, cases in which there was compliance with both antibiotic type and timing had an SSI rate of 3.3% (24 of 718 patients), while those which were non-compliant had a rate of 8.1% (5 of 62) with a *p* = 0.07.

**Table 2:** Characteristics of the Study Cohort Stratified by Surgical Antimicrobial Prophylaxis (SAP) Bundle

Characteristics	Total No. Surgical Procedures (%) *n* = 780	No. SAP Bundle Compliant *n* = 718	No. SAP Bundle Noncompliant *n* = 62	*p*-value*
Sex				
Male	307 (39.4)	280 (39)	27 (43.5)	
Female	473 (60.6)	438 (61)	35 (56.5)	0.48
Age, y				0.07
< 65	547 (70.1)	498 (69.4)	49 (79)	
≥ 65	233 (29.9)	220 (30.6)	13 (21)	
BMI				0.77
< 30	414 (53.1)	380 (52.9)	34 (54.8)	
> =30	366 (46.9)	338 (47.1)	28 (45.2)	
ASA score				0.92
< 3	297 (38.1)	273 (38)	24 (38.7)	
≥ 3	483 (61.9)	445 (62)	4.8 (61.3)	
Diabetes				0.44
Yes	128 (16.4)	120 (16.7)	8 (12.9)	
No	652 (83.6)	598 (83.3)	54 (87.1)	
COPD				0.92
Yes	27 (3.5)	25 (3.5)	2 (3.2)	
No	753 (96.5)	693 (96.5)	60 (96.8)	
Smoker				0.001
Yes	123 (15.8)	104 (14.5)	19 (30.6)	
No	657 (84.2)	614 (85.5)	43 (69.4)	
On steroids				0.15
Yes	44 (5.6)	38 (5.3)	6 (9.7)	
No	736 (94.4)	680 (94.7)	56 (90.3)	
Emergent case				< 0.001
Yes	38 (4.9)	26 (3.6)	12 (19.4)	
No	742 (95.1)	692 (96.4)	50 (80.6)	
Elective case				< 0.001
Yes	624 (80)	594 (82.7)	30 (48.4)	
No	156 (20)	124 (17.3)	32 (51.6)	
SSI				0.07
Yes	29 (3.7)	24 (3.3)	5 (8.1)	
No	751 (96.3)	694 (96.7)	57 (91.9)	

* *p*-value refers to comparison between SAP compliant and SAP noncompliant and are significant at alpha level of 0.05. SSI indicates surgical site infection; ASA, American Society of Anesthesiologists; BMI, body mass index

**Table 3:** Time from Surgical Antimicrobial Prophylaxis (SAP) Administration to Incision (minutes)

Surgical Specialty, No. Surgical Procedures (%)	Mean	Median	p25th	p75th	IQR	SD
General Surgery, 315 (50.4)	28.6	13.0	8.0	24.0	16.0	90.0
Gynecology, 127 (16.3)	17.3	17.0	11.0	22.0	11.0	9.8
Neurosurgery, 95 (12.2)	36.1	18.0	11.0	33.8	22.8	103.7
Orthopedics, 93 (11.9)	41.1	20.0	14.0	32.5	18.5	142.3
Urology, 67 (8.6)	19.2	16.0	8.5	27.0	18.5	13.0
Vascular, 44 (5.6)	22.7	19.0	10.5	28.0	17.5	18.7
Plastics, 15 (1.9)	15.3	18.0	6.0	22.5	16.5	8.6
Thoracic, 15 (1.9)	37.5	14.0	7.5	36.0	28.5	63.8
ENT, 9 (1.2)	15.7	16.0	11.0	20.5	9.5	5.1

P25^th^ = 25^th^ percentile; p75^th^ = 75^th^ percentile; IQR = interquartile range; SD = standard deviation

The constructed neural network is visualized in Fig. 1. The lines represent both positive and negative interactions between the neurodes. There were two hidden layers identified. The first layer had 16 neurodes, while the second layer had eight. It should be noted that there are multiple positive and negative interactions between the neurodes, implying that the occurrence of an SSI is a complex process. The Professional II Plus© ANN models’ results are presented in Table 3. Overall the ANNs predicted SSI with a 69–90% sensitivity and a 60–50% specificity, depending on the variable set used to develop the corresponding ANN model. While use of the bundle compliance variable in the ANN model lead to a higher specificity (i.e., improves predictions of non-SSI occurrence), it leads to lower sensitivity (i.e., reduced the prediction of an SSI occurrence) (Table 4). The sex variable did not affect specificity (i.e., non-SSI predictions), but did reduce the sensitivity for SSI predictions by 10%, though this was still 10% greater than the sensitivity of the model that included the ACS-NSQIP compliance variable.

**Table 4:** Results for ANN Models Predicting Occurrence of SSI

ANN Model (variables used)	SSI prediction sensitivity (*n* = 29)	No SSI prediction specificity (*n* = 617)
All variables including NSQIP compliance variable	69.0%	60.1%
All variables excluding NSQIP compliance variable	89.7%	50.2%
All variables excluding NSQIP compliance and Sex variables	79.3%	50.2%

**Fig. 2:**
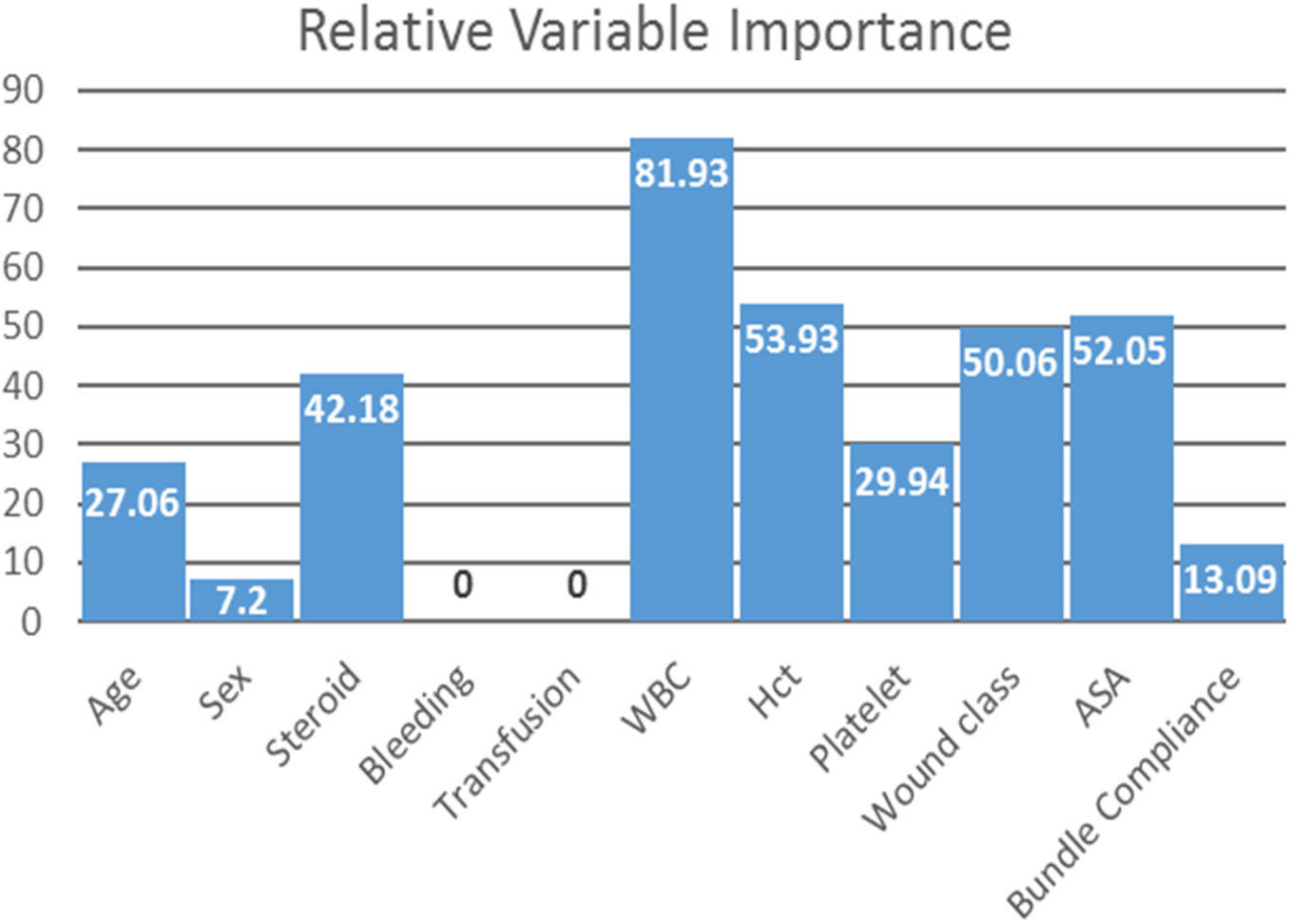
Variable Influence for the ANN Model Using Sum of Connection Weights

The relative variable influence by summed weights, using the JustNN© shell tool, is presented in Fig. 2, with higher values indicating greater significance. The sum of connection weights analysis supports the leave-one-variable-out analysis and indicates that bundle compliance is not a significant variable for predicting SSI occurrence. The most important variable was preoperative WBC count with a value of 82, while the least important one with any value was sex with a value of 6. Bundle compliance was the fourth least important with a value of 13. Figure 2 also indicates that the presence of bleeding disorders and transfusion had no influence whatsoever with values of 0.

## Discussion

What this study demonstrates is that there are complex interactions among the patient factors (such as co-morbidities, medication usage, and physiology as measured by laboratory values) the physician antibiotic ordering practices (such as which antibiotics are ordered), and system factors (such as timing of antibiotic administration) which can affect prediction of an SSI in an individual patient. The complexity of the interaction can clearly be seen in Fig. 1, in which the ANN identified two hidden layers of interactions with a total of 24 neurodes of both positive and negative interactions (Fig. 1). Specifically, the ANN models showed that compliance with a bundle of the appropriate antibiotic type and timing did not show a better sensitivity, although did show a better specificity, in the prediction of the development an SSI compared to the models that did not use this variable. We would like to emphasize that this study is not intended to determine if antibiotic bundle compliance reduces the incidence of SSI occurrences, but rather it has little value in predicting which patient will developed an SSI.

This study has demonstrated that when applied in a broad population of surgical patients and operations, the machine learning nonparametric method of ANNs using supervised backpropagation learning can predict SSI’s with 69 to 90% sensitivity and 60 to 50% specificity, depending on the variable set chosen. The output of the ANN is a prediction for an individual patient about whether they will suffer a SSI following surgery. Higher sensitivity is desirable since this would enable focused monitoring and quicker intervention for patients more likely to suffer an SSI. Nevertheless, the question remains as to why inclusion of the SAP variable decreases the sensitivity of the ANN prediction model by 10 to 20 percentage points, yet improve specificity by about 10 percentage points? We do not have a good answer for this question, but speculate that there is some type of interaction with the previously mentioned patient factors. This will be an area of additional research.

Using a leave-one-variable-out methodology, the two variables NSQIP bundle compliance and sex were evaluated for their contribution to the SSI prediction models. The NSQIP bundle compliance variable was found to not contribute significantly to sensitivity for predicting SSI with a raw 20% and net 30% improvement in sensitivity when this variable was not present, though higher specificity was achieved when the bundle compliance variable was present. A different analysis using a sum of the connection weights methodology was also utilized and confirmed the lack of influence on predicting an SSI (regarding sensitivity) for both NSQIP bundle compliance and sex. Additionally, the sum of the connection weights analysis also indicated that the variables of bleeding disorders and transfusions had no effect.

Several studies have assessed the effectiveness of SSI bundles in reducing SSI occurrences with mixed results. Hawn, et al. [8] and Anthony, et al. [21] report that compliance with an SSI bundle did not reduce SSI rates. Kim, et al. [22] in a systematic review and meta-analysis of laparoscopic cholecystectomy found prophylactic antibiotic reduced superficial SSI’s but not deep SSI’s in low-risk patients. On the other hand, several studies have found benefit in colorectal surgery [23–25], neurosurgery [26], hysterectomy [27], as well as a board national surgical database [9]. In fact, in our study, compliance with the antibiotic type and timing was associated with a lower incidence of postoperative SSI, although it did not quite reach statistical significance. Part of the inconsistency could be because these studies use different bundles [10]; however, part could also be due to the fact that none of their analyses include patient specific factors in the development of SSI’s. Studies of efficacy of bundle compliance have generally focused bundles in the randomized trials or retrospective cohorts, without accounting for individual patient factors. These interactions may affect the predictive value of compliance adherence to SSI development.

There are been numerous studies attempting to develop predictive models for the occurrence of SSI’s. These have included major abdominal surgery [28], spinal surgery [29], vascular surgery [30], ventral hernia [31], and orthopedic surgery [32], among others [33]. However, there has been problems translating these prediction models to other datasets. For example, Bergquist, et al. [34] documented that several colorectal surgery SSI predictive models did not perform as well as their original studies when applied to another institution’s dataset. Yet, studies on SSI prediction have generally focused on patient factors without addressing clinical practice, such as the use of prophylactic antibiotic bundles. Therefore, as with studies assessing efficacy of SSI reduction bundles, SSI prediction models have not assessed fully the interaction of patient factors with actual patient care.

Because the ANN models output a real value, the classification of SSI occurrence was made for all outputs above a pre-specified cutoff value, usually zero. However, modifying the cutoff value enables adjustments in the sensitivity and specificity, but there is a tradeoff. Lowering the cutoff value below zero will improve sensitivity, but at the same time may decrease specificity. Of course, it is pointless to predict that all patients will have an SSI so that no differentiation is performed by the ANN classification. As an example, the third ANN model that did not have both a NSQIP compliance variable and the sex variable had an identical specificity, but the sensitivity was only 79% as opposed to 90% for the model only missing the NSQIP compliance variable. Adjusting the cutoff downwards to make the third ANN model’s sensitivity identical to the ANN model missing only the NSQIP compliance variable resulted in a specificity of 24%. The lower specificity indicates that a greater number of patients who would not suffer an SSI would still need to be monitored for an SSI since they were incorrectly classified as false positives.

ANN is a type of nonparametric machine learning. It searches for “hidden” interactions among the variables which may be associated with the occurrence of a specific event. Other types of nonparametric machine learning programs include support vector machines [35] and conditional inference trees [36]. What we have found in this study is that the most relevant factor was preoperative white blood cell count, while bundle compliance was the fourth least relevant. Others have used machine learning program to predict SSI’s. Ke, et al. [37] used a machine learning algorithm based on temporal changes in wound characteristics to create a model predicting time to SSI development. Soguero-Ruiz, et al. [38] used laboratory testing data with Gaussian process regression, time warping and imputation methods into a support vector machine to predict SSI’s. Interestingly, their analysis, like ours, also showed that platelet count, white blood cell count, hemoglobin level, among others, as relevant factors in their prediction model.

There are several limitations to this study. One limitation is that due to the retrospective nature of the study, not all the recommended NSQIP bundle were well or consistently documented and, therefore, could not be included in the model. It is unknown how including these other factors could have affected the predictive value of compliance with the bundle. Another limitation is that not all possible architectures of ANN were attempted. It is possible that a different architecture could have further improved the sensitivity or the specificity or both for any of the ANN models. Furthermore, ANN modeling has not been applied to other SSI datasets. It is unclear how whether ANN modeling will yield similar results to other datasets. Lastly, we also did not compare our ANN model to experienced practitioners SSI risk assessment for an individual patient. Perhaps such practitioners would be more accurate. Therefore, the results reported should be viewed as the minimum achievable by an ANN SSI prediction model.

Future research is needed to further explore the significance of the 9 variables other than bundle compliance and sex to using the leave-one-variable-out method to further determine their influence on the SSI prediction models. Additional variable may also be evaluated by flipping the approach and adding one in to an existing model and examining the impact on sensitivity and specificity.

## Conclusion

In conclusion, this study has provided evidence for the efficacy of using ANN models to predict SSI. ANN models can achieve almost 90% sensitivity for predicting SSI for a patient. Evidence from the ANN models indicate that knowledge of compliance with SSI reduction protocols is not a factor in predicting likely SSI occurrence. This is not to say that bundle compliance did not reduce the incidence of SSI’s, only that other relevant factors outweigh it as a predictive factor. More importantly, this study is one of the few studies evaluating patient-specific factors as well as a clinical practice factors. Understanding the interaction is a potentially important new frontier in clinical prediction research.

## Data Availability

The datasets used and/or analyzed during the current study are available from the corresponding author on reasonable request.

## Abbreviations

ACS-NSQIP: American College of Surgeons National Surgical Quality Improvement Program
ANN: Artificial neural networks
SAP: Surgical antimicrobial prophylaxis
SCIP: Surgical Care Improvement Program
SSI: Surgical site infection
WBC/mm^3^: White blood count per cubic millimeter
